# Sarkosyl-Induced Helical Structure of an Antimicrobial Peptide GW-Q6 Plays an Essential Role in the Binding of Surface Receptor OprI in *Pseudomonas aeruginosa*

**DOI:** 10.1371/journal.pone.0164597

**Published:** 2016-10-11

**Authors:** Tien-Sheng Tseng, Shih-Han Wang, Ting-Wei Chang, Hung-Mu Wei, Yu-June Wang, Keng-Chang Tsai, You-Di Liao, Chinpan Chen

**Affiliations:** 1 Institute of Biomedical Sciences, Academia Sinica, Taipei, Taiwan; 2 National Research Institute of Chinese Medicine, Ministry of Health and Welfare, Taipei, Taiwan; 3 The Ph.D. Program for Medical Biotechnology, College of Medical Science and Technology, Taipei Medical University, Taipei, Taiwan; University of Pittsburgh School of Medicine, UNITED STATES

## Abstract

The emergence of antibiotic-resistant microbial strains has become a public health issue and there is an urgent need to develop new anti-infective molecules. Although natural antimicrobial peptides (AMPs) can exert bactericidal activities, they have not shown clinical efficacy. The limitations of native peptides may be overcome with rational design and synthesis. Here, we provide evidence that the bactericidal activity of a synthetic peptide, GW-Q6, against *Pseudomonas aeruginosa* is mediated through outer membrane protein OprI. Hyperpolarization/depolarization of membrane potential and increase of membrane permeability were observed after GW-Q6 treatment. Helical structure as well as hydrophobicity was induced by an amphipathic surfactant, sarkosyl, for binding to OprI and possible to membrane. NMR studies demonstrated GW-Q6 is an amphipathic α-helical structure in DPC micelles. The paramagnetic relaxation enhancement (PRE) approach revealed that GW-Q6 orients its α-helix segment (K7-K17) into DPC micelles. Additionally, this α-helix segment is critical for membrane permeabilization and antimicrobial activity. Moreover, residues K3, K7, and K14 could be critical for helical formation and membrane binding while residues Y19 and W20 for directing the C-terminus of the peptide to the surface of micelle. Taken together, our study provides mechanistic insights into the mode of action of the GW-Q6 peptide and suggests its applicability in modifying and developing potent AMPs as therapeutic agents.

## Introduction

The progressive increase of antibiotic-resistant bacterial strains has become a severe public-health problem worldwide [1,2]. There is an urgent need to develop new antibiotics with less likelihood to incur evolved resistance. Animals evolutionarily acquire innate abilities from ancestors to identify and resist attacks by microorganisms. The immune resistance is correlated with the development of a specific immune response. Antimicrobial peptides (AMPs) are natural components of the innate immune system for the majority of living organisms ranging from prokaryotes to humans [3–5]. These peptides can kill microbial cells by targeting and disrupting the plasma membrane and have been reported to exhibit minimal inhibitory concentration against bacteria in the micromolar range [6–8]. As a result, AMPs are attractive candidates for development into novel and potent therapeutics for infections caused by multidrug-resistant bacteria.

To date, a large amount of characterized AMPs can be found in the Antimicrobial Peptide Database (http://aps.unmc.edu/AP/main.php) which serves as a platform to predict the structure, function, and antimicrobial activity of any queried sequence [9–11]. AMPs are generally less than 50 amino acids in length with positive charges ranging from +2 to +9 and a large collection of hydrophobic residues [7,12,13]. Natural AMPs are structurally categorized into four main groups: loops, α-helices, β-sheets, and extended peptides with α-helices and β-sheets being the most common [13,14]. These various AMPs share two important functional requirements: (a) a net cationicity‒AMPs exert their cell lytic ability by first binding to the negatively charged microbial surface and (b) the ability to assume an amphipathic structure‒a large number of AMPs are unstructured and linear in solution. However, upon binding and/or inserting into the target membrane, they transform into amphipathic helices [13,15–19]. This further breaks down the transmembrane potential and disrupts the balance of ion gradients resulting in leakage of cell contents and eventual cell death.

AMPs should demonstrate potent antimicrobial abilities and low hemolytic activities if they were to be developed into therapeutics. It has been reported that high amphipathicity and hydrophobicity of AMPs are correlated with increased hemolytic activity [20]. Nevertheless, naturally occurring AMPs have low bioavailability and are susceptible to degradation by proteases [21,22]. Therefore, peptides with improved and shorter amino acids sequences have been made to increase bactericidal activity while decreasing hemolytic activity and cytotoxicity [23–26]. Recently *Chou et al*. designed a series of cationic α-helical peptides based on structural parameters, charge, polar angle, hydrophobicity and hydrophobic moment [20,27]. Their synthetic cationic AMPs named GW-Q4, GW-Q6, GW-H1, and GW-M1 showed enhanced antimicrobial ability, decreased hemolytic activity, and improved selectivity over two potent natural AMPs named magainin 2a and pleurocidin. However, the bacterial targets of these AMPs and their mode of action are hitherto unclear. Our previous study showed that the outer membrane protein OprI plays a vital role in the susceptibility of the Gram-negative bacteria *Pseudomonas aeruginosa* to cationic α‒helical AMPs. OprI could maintain the integrity of the outer membrane of microbes and serve as the receptor for cationic α‒helical AMPs [28,29]. Here we find that the bactericidal activity of the GW-Q6 peptide is potentially associated with the OprI receptor. This focuses our attention on the mode of action of GW-Q6 by studying the membrane potential and permeability of the bacterium. Here, we analyzed the structural properties of GW-Q6 in hydrophobic conditions, its solution structure bound with membrane-mimetic micelles, and investigated its interaction with the OprI receptor.

In this report, we investigated the GW-Q6 peptide’s mechanism of action by molecular, biological, biochemical, and biophysical methodologies. Change in membrane potential and permeability as well as enhanced binding of GW-Q6 to the membrane receptor OprI by the amphiphilic surfactant sarkosyl were observed. Furthermore, CD, dye-leakage fluorescence assay, two-dimensional NMR, spin labeling NMR, and MD simulation experiments were performed to approximate the secondary structure, dye-leakage activity, solution structure, orientation in DPC micelles, and peptide-micelle model of GW-Q6 respectively. These structural and functional insights present valuable information for further modification or development of new anti-infective agents.

## Materials and Methods

### Materials

GW-Q6 peptide and biotinylated GW-Q6 (GIKIAKKAITIAKKIAKIYW) were synthesized by Kelowna International Scientific Inc., Taipei, Taiwan, with more than 95% purity and their molecular sizes were verified by mass spectrum analysis. 1-palmitoyl-2-oleoyl-*sn*-glycero-3-phosphocholine (POPC) and 1-palmitoyl-2-oleoyl-*sn*-glycero-3-phosphoglycerol (POPG) were purchased from Avanti Polar Lipids, Inc. Calcein, MnCl_2_, 5-, 12-, 16-doxyl stearic acids, and 8-anilino-1-naphthalenesulfonic acid (ANS) were purchased from Sigma-Aldrich Inc. Dodecylphosphocholine-*d*_*38*_, methanol-*d*_*4*_ and D_2_O were supplied from Cambridge Isotope Laboratories, Inc. Sodium dodecyl sulfate was obtained from Merk (Darmstadt, Germany). Streptavidin gel was purchased from GE Healthcare (Uppsala, Sweden). Sodium N-dodecanoylsarcosinate (sarkosyl) was supplied by Wako Pure Chem (Osaka, Japan). 1-ethyl-3-[3-dimethylaminopropyl] carbodiimide hydrochloride (EDC) was purchased from ThermoFisher (St. Waltham, MA, USA).

### Circular Dichroism (CD) Spectroscopy

CD spectra were acquired with an Aviv CD 202 spectrometer (Lakewood, NJ). GW-Q6 was dissolved in 10 mM sodium phosphate (pH 5.0) to yield a 2 mM stock. CD samples: GW-Q6 (60 μM) in 20, 30% TFE, GW-Q6: DPC = 1: 100 (molar ratio), and GW-Q6: SDS = 1: 100 were prepared by diluting the GW-Q6 peptide stock (2 mM) into TFE, DPC, and SDS individually to achieve the final concentrations. In addition, GW-Q6 (60 μM) was also prepared in PC buffer (20 mM Hepes, pH 7.4, 0.05 M NaCl) and sarkosyl solution (10 mM sodium phosphate, pH 7.4, 0.15 M NaCl, 0.075% sodium N-dodecanoylsarcosinate) for CD experiments. The CD spectra were recorded at 25°C with wavelength ranges between 260 and 190/200 nm using a 1-mm path length quartz cuvette. All spectra were averaged over three scans and converted to mean residue ellipticity [θ]. The helical content of individual samples was evaluated using the BESTSEL (http://bestsel.elte.hu/).

### Preparation of large unilamellar vesicles (LUVs)

The preparation of large unilamellar vesicles was generated by the extrusion method as reported by *Wei et*. *al* [30]. The phospholipids, 1-palmitoyl-2-oleoyl-*sn*-glycero-3-phosphocholine (POPC) and 1-palmitoyl-2-oleoyl-*sn*-glycero-3-phosphoglycerol (POPG), were dissolved in chloroform and completely dried by nitrogen air. Subsequently, the prepared dried lipid film was dissolved in PBS buffer (137 mM NaCl, 2.7 mM KCl, 10 mM Na_2_HPO_4_, and 1.8 mM KH_2_PO_4_, pH 7.4) by vortexing, and underwent the freezing and thawing cycles 10 times. Furthermore, the lipid suspensions were extruded by an mini-extrusion device (Avanti Polar Lipids, Inc., Alabaster, AL, USA) through two staked 0.4 μm-pore-size polycarbonate filters 10 times, and then subjected to another extrusion with two stacked 0.1 μm-pore-size filters for another 10 times to generate the LUVs. Likewise, the calcein-entrapped LUVs were generated in calcein-containing buffer (70 mM calcein and 10 mM Tris at pH 7.4) with the same process as previous. Unentrapped calcein was eliminated by centrifugation (10,000 rpm for 10 min) three times using isosmotic buffer (10 mM Tris and 100 mM NaCl, pH 7.4). Finally, the size of the generated LUVs were confirmed by dynamic light scattering (DLS) on a Zetasizer Nano ZS (Malvern Instruments, Malvern, UK).

### Calcein leakage assay

Peptide-induced calcein leakage as shown by an increase in fluorescence was conducted with a JASCO FP-8500 spectrofluorometer (JASCO, Tokyo, Japan) at an excitation and emission wavelengths of 496 and 515 nm. Measurements were made in ~30 μM lipids of calcein-entrapped LUVs in 20 mM Tris and 100 mM NaCl at pH 7.4 at 25°C. 100% leakage was induced in 3 min by the addition of 0.1% (v/v) Triton X-100. The degree of leakage induced by various concentration of peptides was calculated using the following equation: % leakage = [(*F* − *F*0)/(*Fr* − *F*0)] × 100

where *F0* and *Fr* are the initial fluorescence intensities observed without peptide and after treatment of Triton X-100.

### Antimicrobial activity assay

Bacteria were cultured in Luria-Bertani broth and plated on Luria-Bertani agar for *Pseudomonas aeruginosa* PAO1 (ATCC BAA-47TM), *P*. *aeruginosa* (ATCC 27853), *Klebsiell*a *pneumoniae* (ATCC 13884), and *Staphylococcus aureus* MRSA (ATCC49476). Vancomycin-resistant enterococcus clinical isolate VRE 2061007 from National Taiwan University Hospital were cultured and plated in/on tryptic soy broth/agar (Difco0369). *Listeria monocytogenes* was cultured in/on Bacto^TM^ BHI broth/agar (BD). The microbes were grown overnight, washed, and diluted 1:300 in 10 mM sodium phosphate, pH 7.4. 45 μl of the microbes (5–10 x10^4^ colony forming units (cfu)) were mixed with serially diluted GW-Q6 (5 μl) and incubated at 37°C for 1.5 hrs. Serial dilution of each AMPs-treated bacteria was prepared and plated for the determination of the remaining cfu [28]. At least three independent experiments were performed for each assay to determine the average value with standard deviation. Alternatively, the bactericidal activity of GW-Q6 against higher concentration of *P*. *aeruginosa* PAO1 (5 x10^6^ cfu/45μl) was performed in equilibrium buffer (5 mM Hepes, pH 7.2, 20 mM glucose, 0.2 mM EDTA, and 0.1 M KCl) which was used for determination of membrane potential [31].

### Membrane potential and permeability assays

*P*. *aeruginosa* PAO1 cells were collected from mid-log-phase culture, washed in Hepes buffer (5 mM Hepes, pH 7.2, and 20 mM glucose), and re-suspended in the same buffer (2 x10^7^cfu/200μl) with the addition of 0.2 mM EDTA. The bacteria were incubated with 0.4 μM DiSC_3_(5) (3,3’-dipropylthiadicarbocyanine iodide, Molecular Probes, OR, USA) in the dark for 2 hrs at room temperature with gentle agitation (150 rpm). The osmotic gradient was equilibrated to a final concentration of 0.1 M KCl. The GW-Q6 peptides were added to the above cell suspension in a High Precision Cell cuvette (Hellma Analytics, Mülheim, Germany). The fluorescence intensity was determined by an FP-8500 fluorescence spectrophotometer (Jasco, Tokyo, Japan) with an excitation wavelength of 622 nm and an emission wavelength of 670 nm [31]). Microbes were then collected, washed, and re-suspended in distilled water (2–5 x10^7^cfu/100μl) to test for permeability. 1.0 μM SYTOX^R^Green (Molecular Probes, OR, USA) was added into cell suspension in a 96-well plate for 5 min in the dark before addition of GW-Q6. The fluorescence intensity of SYTOX^R^Green bound to cytosolic DNA was determined by a SpectraMax M2 microplate reader (Molecular Devices, CA, USA) with an excitation wavelength of 485 nm and an emission wavelength of 520 nm) [32].

### Cross linking

Small aliquots of bacterial suspension (5–10 x10^6^ cfu in 45 ul) which were previously treated with GW-Q6 were incubated with 25 mM EDC in 10 mM sodium phosphate, pH 5.5, at 37°C for 30 min and subjected to SDS-PAGE and Western blot analysis using an anti-OprI antibody [28].

### Binding of OprI by biotinylated AMPs

Streptavidin-conjugated beads were incubated with biotinylated GW-Q6 peptide in PC buffer (20 mM Hepes, pH 7.4, 0.05 M NaCl), or sarkosyl solution (10 mM sodium phosphate, pH 7.4, 0.15 M NaCl, 0.075% sodium N-dodecanoylsarcosinate) for 2 hrs at 4°C on a rolling wheel. The immobilized biotinylated GW-Q6 peptide was further mixed with recombinant OprI overnight at 4°C, washed three times with respective buffer, and subjected to non-reducing SDS-PAGE/Coomassie blue staining. The preparation of recombinant OprI was described as mentioned previously [33].

### Measurement of ANS fluorescence

The emission spectra of 8-anilino-1-naphthalenesulfonate (ANS) excited at 380 nm were measured between 400 and 600 nm at 20°C using a temperature-controlled JASCO FP-8500 spectrofluorometer (JASCO, Tokyo, Japan) [33]. A small volume of ANS stock solution was added to a 200 μl PC buffer containing 80 μg/ml OprI, and/or 10 μg/ml GW-Q6 at a concentration of 10, 20, 30, 40 and 50 μM. In addition, the ANS emission spectrum of GW-Q6 was also measured while in sarkosyl solution.

### NMR spectroscopy

The NMR samples were prepared by mixing GW-Q6 (final concentration = 1.5 mM) with DPC (final concentration = 150 mM) to reach a molar ratio of 1:100, consisting of 10 mM sodium phosphate and 10% D_2_O at pH 5.0. The pH values of samples were adjusted before NMR measurements. All NMR experiments were conducted on a Bruker Avance 600 and 800 MHz spectrometers at 320 K. 2D-NOESY spectra were acquired at two distinct mixing times of 150 and 300 ms. TOCSY spectrum was recorded with mixing times of 60 ms at 2048 points in *t*2 and 320 points in *t*1. Spectral data were processed using TopSpin 3.1 (Brucker Spectrospin), NMRPipe [34] and Sparky (T. D. Goddard and D. G. Kneller, University of California, San Francisco).

### Structure calculation

2D-NOESY spectrum (150 ms mixing time) of GW-Q6 peptide in DPC micelles (molar ration = 1:100) was recorded at pH 5.0, 320 K to obtain distance constraints by manually assigning NOE cross-peaks (Table 1). Based on the peak intensity, the NOE cross-peaks are classified into strong, medium, and weak, which correspond to distance range of 1.8–2.8 Å, 1.8–3.4 Å, and 1.8–5.0 Å, respectively. The backbone dihedral angle constraints were derived from DQF-COSY spectrum [35]. The solution structures were generated by using CNS 1.2 for restrained molecular dynamic simulations [36,37]. The final ensemble contained 15 lowest energy structures and was evaluated using PROCHECK-NMR [38] and MOLMOL [39]. PyMol (http://www.pymol.org) was used for molecular visualization.

**Table 1:** NMR Structure Calculation Parameters.

**NOE restraints**	
Intraresidue NOEs	140
Sequential NOEs [(i-j) = 1]	122
Medium-range NOEs (|i-j| ≤ 4)	121
Total NOEs	383
Dihedral angle restraints	18
**Ramachandran plot summary (%)**	
Most favored	87.9
Additionally allowed	10.6
Generally allowed	1.5
Disallowed	0
**Average RMSD from mean structure**	
Back atoms	0.33
All heavy atoms	0.49

## Results

### Membrane-permeabilizing ability of GW-Q6 against synthetic LUVs

**Fig 1:**
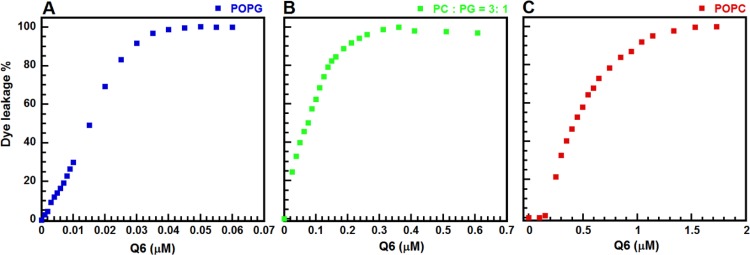
Calcein leakage caused by GW-Q6 peptide against POPC, POPG, and POPC/POPG (3: 1) LUVs. (**A**) Profile of calcein leakage as a function of GW-Q6 concentration for POPG LUVs. (**B**) Representation of calcein leakage as a function of GW-Q6 concentration for POPC/POPG (3: 1) LUVs. (**C**) Demonstration of calcein leakage as a function of GW-Q6 concentration for POPC LUVs.

The membrane-permeabilizing ability of the GW-Q6 peptide was estimated and quantified by the leakage of calcein entrapped in LUVs having different surface charges. The highly negative charged POPG as well as negatively charged mixed POPC/POPG (3: 1) LUVs were used to mimic the bacterial membrane, and the neutral POPC LUVs were utilized to mimic eukaryotic membranes. The dye leakage activity (LC_100_) is defined as the minimal concentration of GW-Q6 to cause 100% leakage of calcein from the LUVs. GW-Q6 exerted significant leakage of POPG LUVs with LC_100_ of 0.045 μM and the LC_100_ for POPC/POPG (3: 1) LUVs was ~ 0.36 μM as shown in Fig 1A and 1B. On the contrary, GW-Q6 exhibited weaker membrane-disrupting ability against POPC with LC_100_ ≥ 1.5 μM. These results suggest that GW-Q6 has a strong disrupting activity against negatively charged bacterial membranes but displays much weaker ability against neutrally charged eukaryotic cell membranes [40].

### Effect of GW-Q6 on the membrane potential and permeability of bacteria

**Fig 2:**
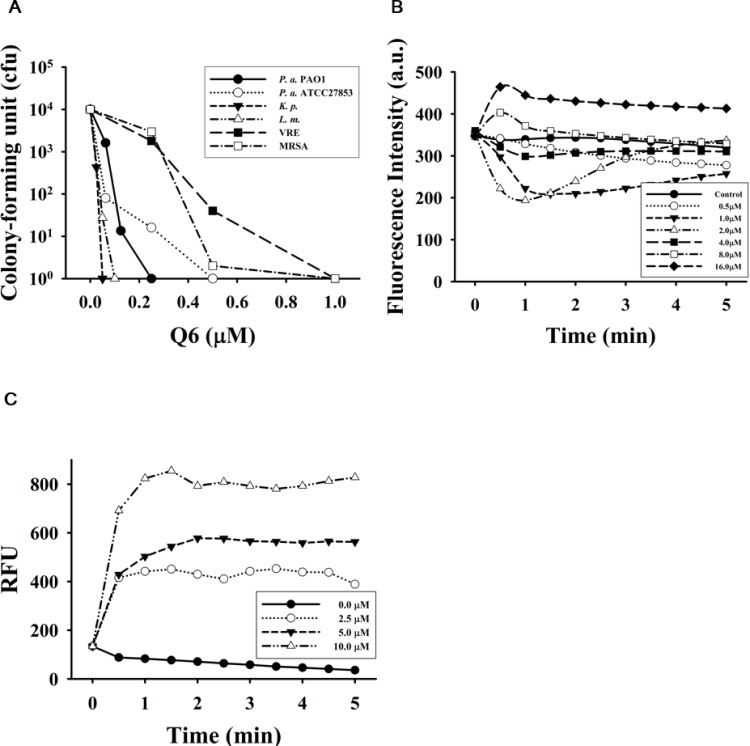
Bactericidal activity attributed to GW-Q6 peptide. (**A**) Antimicrobial spectrum of GW-Q6 against Gram-positive and -negative bacteria. The overnight cultures of microbes were diluted (1:300) in 10 mM sodium phosphate, pH 7.4. Small aliquots of bacterial suspension (45 μl) were incubated with various concentrations of GW-Q6 (5μl) at 37°C for 1.5 hrs and plated for the determination of remaining cfu. (**B**) Membrane potential of GW-Q6-treated *P*. *aeruginosa* was determined in the presence of DiSC_3_ (5). Fluorescence intensity was monitored at an excitation wavelength of 622 nm and an emission wavelength of 670 nm. Data plots are representative average values of three independent trials measured in absorbance unit (a.u.). (**C**) Membrane permeability of *P*. *aeruginosa* was monitored in the presence of SYTOX^TM^ Green at 485 nm and 520 nm for excitation and emission wavelength. Data plots are normalized with values of untreated sample and representative average values of three independent trials. Abbreviations are defined for RFU, relative fluorescence unit; *P*.*a*.*; Pseudomonas aeruginosa; K*.*p*., *Klebsiella pneumoniae*; *L*.*m*., *Listeria monocytogenes*; VRE, vancomycin-resistant enterococcus; MRSA, *Staphylococcus aureus* MRSA.

The GW-Q6 peptide exerted a broad antimicrobial spectrum. The Gram-negative bacterium *Klebsiella pneumoniae* was the most sensitive among all of the microbes tested in 10 mM phosphate buffer (0.01 μM GW-Q6 for 10^2^-fold reduction in cfu compared with that of buffer only) and thereafter in the order of Gram-positive *Listeria monocytogenes* (0.03μM), Gram-negative *Pseudomonas aeruginosa* ATCC27853 (0.06 μM) and *P*. *aeruginosa* PAO1 (0.1 μM). Whereas the Gram-positive *Staphylococcus aureus* MRSA (0.35 μM) and vancomycin-resistant enterococcus clinical isolate VRE (0.42 μM) bacteria were less sensitive (Fig 2A). Similar to most AMPs, the GW-Q6 peptide induced depolarization of bacterial membrane potential because the DiSC_3_(5) dye was released into the surrounding medium that caused an increase of fluorescence intensity at higher concentrations (8 μM and 16 μM). However, hyperpolarization was observed in the first few minutes after GW-Q6 treatment and was turned back to neutral condition subsequently at lower concentrations (1 μM and 2 μM) (Fig 2B). It is worthy to mention that 4 μM of GW-Q6 peptide was able to cause 10^2^-fold reduction in cfu in assay condition with equilibrium buffer (data not shown). The membrane permeability of the bacteria markedly increased in minutes after 2.5–10 μM GW-Q6 treatment (Fig 2C). These results suggest that GW-Q6 employs a unique membrane-permeabilizing pathway different from those of conventional AMPs.

### Involvement of OprI in the susceptibility of *P*. *aeruginosa* to GW-Q6

**Fig 3:**
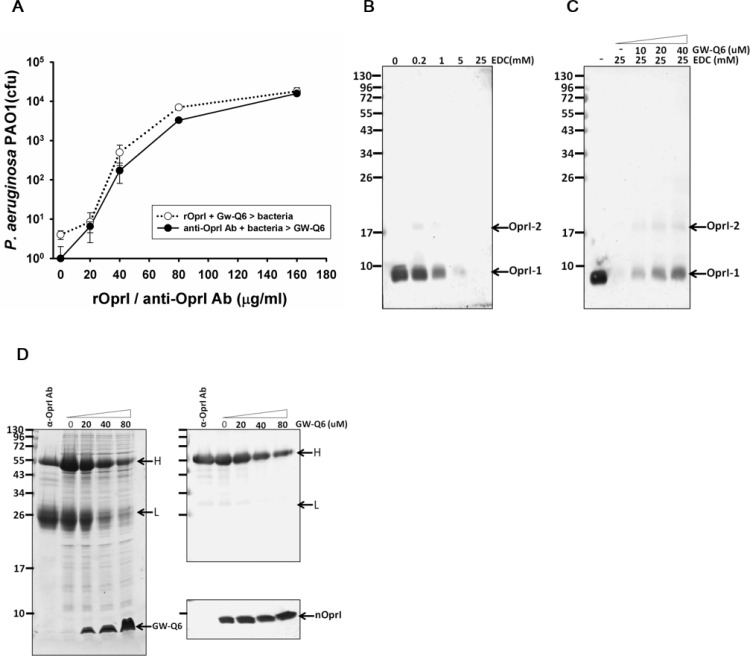
Role of OprI in the susceptibility of *P*. *aeruginosa* to the GW-Q6 peptide. (**A)** Repression of bactericidal activity of GW-Q6. The recombinant OprI and anti-OprI antibody were pre-incubated with GW-Q6 and bacteria for 30 min before adding to bacteria and GW-Q6, respectively. (**B-C**) Analysis of OprI after EDC cross-linking. Small aliquots of control and GW-Q6-treated *P*. *aeruginosa* were suspended in 10 mM sodium phosphate, pH 5.5, crosslinked with EDC as indicated and subjected to reduced SDS-PAGE and Western blotting using anti-OprI antibodies. (**D**) Binding of anti-OprI antibody to bacteria. 10 μg of Protein-A purified anti-OprI antibody was added to control and GW-Q6-treated *P*. *aeruginosa*. The resultant pellets were subjected to non-reducing SDS-PAGE followed by Coomassie blue staining (left panel) and Western blotting for immunoglobulin and OprI (right panel). nOprI-1 and nOprI-2 represent monomeric and dimeric native OprI. H and L represent heavy and light chains of immunoglobulin.

The antimicrobial activity of GW-Q6 against *P*. *aeruginosa* PAO1 was significantly repressed by the presence of exogenous recombinant OprI as well as anti-OprI antibodies, which was employed to compete for GW-Q6 or block surface OprI, respectively (Fig 3A). The surface hexameric OprIs in association with nearby components were accessible to exogenous crosslinking agent. Thus the amount of monomeric OprI was significantly decreased as a function of EDC concentration as detected by SDS-PAGE and Western blotting (Fig 3B). In contrast, when the bacterium was pre-treated with GW-Q6, OprI became inaccessible to EDC and dissociated into monomers (Fig 3C). The control bacteria were well recognized by the anti-OprI antibody, but became less susceptible after GW-Q6 treatment. Of particular note is that the amount of OprI remained constant in a dose-dependent manner with GW-Q6 treatment (Fig 3D). These results suggest that the OprI receptor on the bacterial surface was targeted by GW-Q6 and may be internalized into cytosol.

### Sarkosyl-induced conformational change of GW-Q6 leading to the increase of OprI-binding

**Fig 4:**
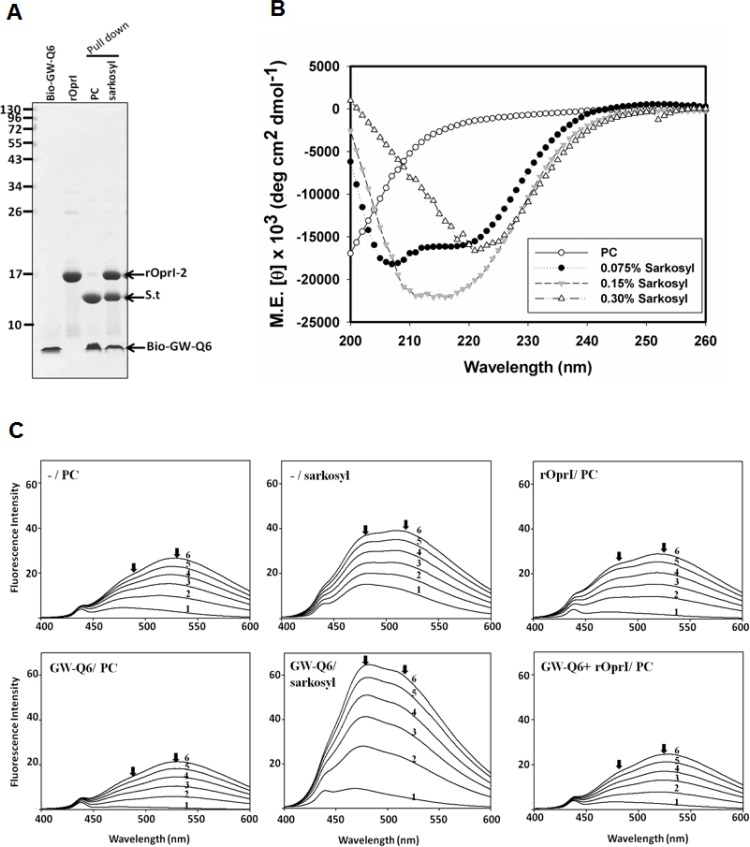
GW-Q6 peptide conformational change and enhancement of OprIbinding by sarkosyl. (**A**) Enhancement of OprI- binding to GW-Q6 by sarkosyl. Biotinylated GW-Q6 was immobilized on streptavidin-conjugated beads in PC buffer or sarkosyl solution, then further incubated with recombinant OprI, and subjected to non-reducing SDS-PAGE/Coomassie blue staining. (**B**) CD spectrum of GW-Q6 in PC buffer and sarkosyl solutions containing different concentration of sarkosyl as indicated. (**C**) ANS emission spectra of GW-Q6. The emission spectra of ANS in the presence of 20 μg/ml GW-Q6 and/or 80 μg/ml rOprI were measured in 200 μl solution. ANS was added and adjusted to the indicated concentrations (lines 1 to 6 at 0, 10, 20, 30, 40 and 50 μM, respectively). Arrows indicate the wavelength emission maximum at 470 nm or 520 nm by bound- and free-form ANS. rOprI represents recombinant OprI.

The CD spectrum of GW-Q6 exhibits a typical random coil conformation in 10 mM sodium phosphate, pH 5.0. However, GW-Q6 in 20% and 30% TFE appeared to be in μ-helical conformation (α-helical content are 19.7% and 33% respectively) (S1 Fig). Additionally, with a peptide to SDS molar ratio of 1:100, GW-Q6 is more helical (α-helical content 42.8%) than in 30% TFE. When subjected to DPC micelles (peptide to DPC molar ratio = 1:100), GW-Q6 showed nearly identical α-helical content (43.5%) with that in SDS. Notably, the biotinylated GW-Q6 recognized and bound to recombinant OprI only in the sarkosyl solution containing 0.075% sarkosyl (2.6 mM), and rarely in PC buffer (Fig 4A), but not at higher concentration of sarkosyl (0.15%, or 0.3%) (data not shown). Interestingly, the binding was not observed in the presence of other surfactants, like Tween 20, Triton X-100 and SDS at the same concentration as that of sarkosyl. The positive effect of sarkosyl on the binding of GW-Q6 to OprI was further analyzed by CD as well. Similar to the binding results, the GW-Q6 has an apparent α-helical conformation only in the solution containing 0.075% sarkosyl, but not at higher concentration of sarkosyl (0.15%, or 0.3%) (Fig 4B). The conformational changes induced by sarkosyl was further verified by ANS (8-anilino-1-naphthalenesulfonate) fluorescence assay. ANS probes the hydrophobic regions of peptide/protein interface‒the free form ANS exhibits an emission maximum at 520 nm; bound form of ANS mainly emits at 470 nm (blue shift). The ANS spectrum showed that GW-Q6 underwent the blue shift (from 520 to 470 nm) in sarkosyl buffer, in comparison with those of peptides in PC buffer (Fig 4C). These results indicate that sarkosyl induced the increasing hydrophobicity of α-helical GW-Q6, leading to the enhancement of binding to OprI receptor and possibly to the membrane.

### Solution structure of GW-Q6 in complex with DPC micelle

**Fig 5:**
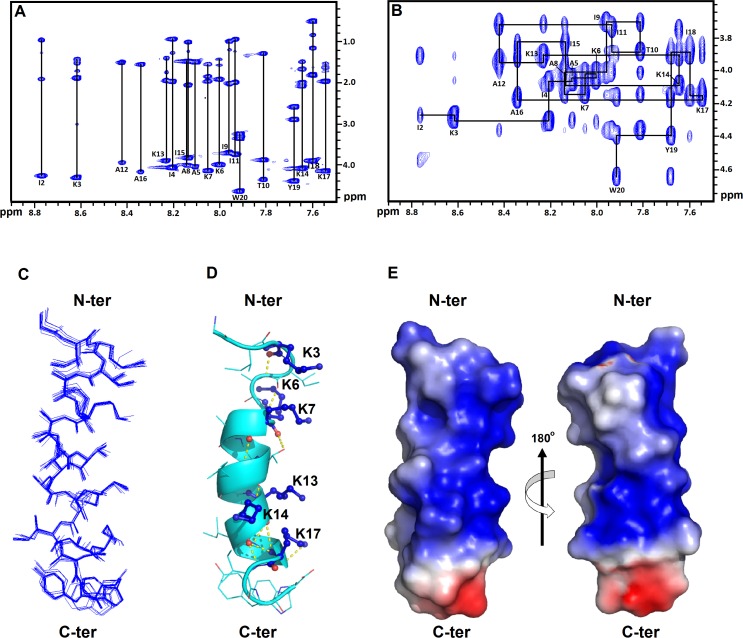
NMR spectra and solution structure of GW-Q6 in DPC micelles at pH 5.0 and 320K. (**A**) A 800-MHz TOCSY spectrum recorded at 60 ms. (**B**) The finger-print region of the NOESY spectrum (mixing time = 150 ms). In (**A**) and (**B**), peaks are labeled at the positions of the NH-C_α_H cross-peaks. (**C**) Superposition of the 15 lowest energy structures of GW-Q6. The backbones and heavy chains are shown in blue lines. (**D**) Ribbon representation of the averaged DPC-bound solution structure. Lysine residues colored in blue are shown as ball-and-sticks. (**E**) The electrostatic surface of DPC-bound GW-Q6. The positive charge is shown in blue; negative charge in red; and neutral positions are colored in white.

NMR analysis was performed to further investigate the atomic structure of the GW-Q6 peptide. The cross peaks of both 2D-TOCSY and 2D-NOESY were well dispersed in DPC at pH 5.0 and 320 K (Fig 5A and 5B). However, in the presence of negatively charged SDS micelles and 0.075% sarkosyl, high concentration of GW-Q6 (1.5 mM) showed aggregation and exhibited poorly dispersed and low resolved spectra (data not shown). As a result, the solution structure of GW-Q6 was determined in DPC micelles. The sequential assignments were achieved by employing 2D-TOCSY and NOESY spectra. 383 NOE-derived distance constraints, consisting of 140 intra-residues, 122 sequential, and 121 medium range distance restraints, combined with 18 backbone dihedral angles were used to calculate the micelle-bound GW-Q6 structure (Table 1). An overlaid ensemble of 15 lowest energy structures is shown in Fig 5C. The rmsd calculated from the averaged coordinate was 0.33 Å for backbone heavy atoms and 0.49 Å for all heavy atoms (Table 1). In nearly all structures, residues K7-K17 were helical. Moreover, Ramachandran plot analyzed by PROCHECK demonstrated that 87.9%, 10.6%, and 1.5% of residues are located in the most ideal regions. The detailed structural statistic is presented in Table 1. The overall structure of GW-Q6 consists of a three-turn α-helix with the loop conformation observed in both the N- and C-termini (Fig 5D). The structure of the GW-Q6 peptide is amphipathic t with the hydrophilic side chains of lysine residues (K3, K6, K7, K13, K14, and K17) on one side and the hydrophobic side chains of the remaining residues are on the other side (Fig 5D). Such an amphipathic helix, rich in positively charged side chains (Fig 5E), is attractive to negatively charged bacterial membranes.

### Localization of GW-Q6 in DPC Micelle

**Fig 6:**
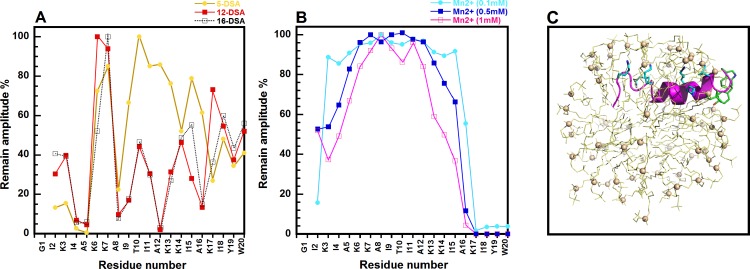
The remaining amplitude and model structure of GW-Q6 in DPC micelles. (**A**) The remaining amplitudes of NH-C_α_H cross peaks of GW-Q6 due to 5-DSA (colored in yellow), 12-DSA (colored in red), and 16-DAS (colored in black), respectively. The concentration of each DSA corresponds to a spin label per micelle. (**B**) The remaining amplitudes of cross peaks of GW-Q6- NH-C_α_H in the presence of 0.1 mM (colored in cyan), 0.5 mM (colored in blue), and 1.0 mM (colored in magenta) MnCl_2_, individually. (**C**) The model structure of GW-Q6 in complex with DPC micelle deduced from PRE results. The GW-Q6 peptide was shown as magenta ribbon with lysine residues and I18, Y19, and W20 presented in cyan and green sticks. The DPC lipids were displayed as light-yellow lines with the phosphorus atoms shown as spheres.

The position of the GW-Q6 peptide in DPC micelles was investigated by using paramagnetic relaxation enhancement (PRE) experiments (see supporting information for detail). Paramagnetic lipids, 5-DSA, 12-DSA, and 16-DSA were used to probe the localization of GW-Q6 in the DPC micelles. The spin-labeled fatty acids potentially broadened NMR signals by enhancing the *T*_*2*_ transverse relaxation rate of protons in close contact to doxyl-stearic acid [41]. 5-DSA, 12-DSA, and 16-DSA have the paramagnetic doxyl-group at the fifth, twelfth, and sixteenth carbon position of acyl chain, respectively. 5-DSA perturbs the NMR signals of NH-CαH which are near the 3–4 atom positions with regard to the site of spin label or close to the surface of the micelle. Likewise, 12- and 16-DSAs affect the NMR signals of NH-CαH which are inserted or buried deeply into the micelle. In this study, the perturbations of NMR NH-CαH cross-peak intensities, upon adding the paramagnetic lipids, were evaluated by TOCSY spectra. The addition of 5-, 12-, and 16-DSA to the GW-Q6 conjugating with micelle reveals the attenuation of intensities for the NOE peaks (Fig 6). The cross peaks of NH-CαH resonances in TOCSY were used to analyze the relative intensity decrease compared to the peptide in DPC micelle without spin label effects. The apparent disturbances in peak intensities of residues (I2-A16) with the addition of 5-, 12- and 16-DSA demonstrate that the GW-Q6 peptide potentially interacts with DPC micelle (Fig 6A). Residues I2 and K3 were mainly affected by 5-DSA, indicating their close proximity with the head group of DPC. Additionally, residues A8-A16 were mostly perturbed by 12- and 16-DSA, suggesting that the α-helical segment is buried and inserted into the DPC micelles. However, residues K17-W20 showed less perturbation upon the additions of DSAs. To precisely elucidate the position of C-terminal residues K17-W20, the PRE experiments were conducted with the addition of 0.1, 0.5 and 1 mM Mn^2+^ ions. Mn^2+^ ion associated with the phosphates in the polar head group of DPC and would broaden the NMR resonance and decrease the intensities of the nearby nucleus. The results showed that the peak intensities of residues K17-W20 were significantly reduced in the presence of 0.1, 0.5, and 1 mM Mn^2+^ ions (Fig 6B). This suggest that the C-terminus is most likely located outside or on the surface of the DPC micelles.

### Molecular Dynamic Simulation of GW-Q6 in DPC Micelles

**Fig 7:**
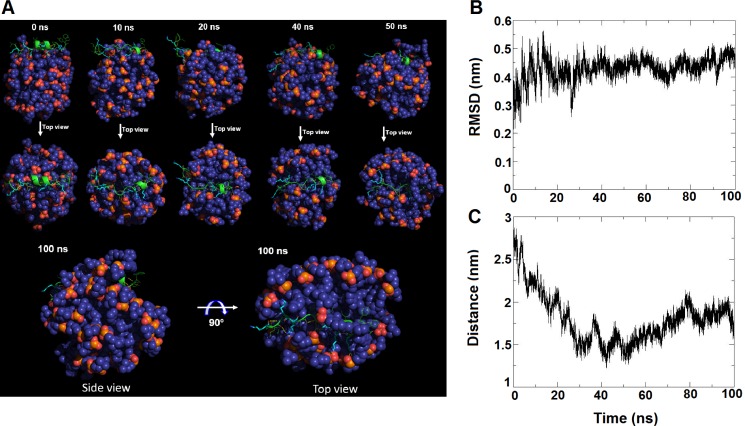
Molecular dynamic simulation of GW-Q6 in DPC micelles. (**A**) A picture of GW-Q6 interacting with DPC micelle is presented as snapshots at 0, 10, 20, 40, 50, and 100 ns simulation times. The DPC lipid is presented as spheres colored in deep blue and the GW-Q6 peptide is shown in green ribbon with lysine residues colored in cyan. (**B**) GW-Q6 structure versus simulation time as quantified by backbone RMSD to NMR structure. (**C**) GW-Q6 position versus simulation time as quantified by distance from peptide backbone center-of-mass to micelle acyl chain center-of-mass.

The PRE result demonstrates the position of GW-Q6 peptide in the DPC micelles, but does not provide a detailed interactive picture between peptide and DPC micelle. Therefore, MD simulation was performed to provide a more in depth evaluation of GW-Q6-micelle interaction (see supporting information for detailed MD setting). First, the pre-determined solution structure of GW-Q6 was positioned at the solvent/hydrophobic interface of the DPC micelle in the initial step of simulation. The conformational drift of GW-Q6 upon binding to DPC micelle was monitored by calculating the RMSD of the backbone atoms during simulation (Fig 7B). The backbone RMSD fluctuated between 2.2–4.3 Å during the 100 ns simulation period. The overall picture of how GW-Q6 interacted with DPC micelles as a function of simulation time was presented in Fig 7A. The GW-Q6-micelle complex obtained at the 10 ns frame showed a less compact structure with a recognizable helix between residues A11-K17. In this conformation, GW-Q6 anchored onto the surface of DPC micelles mainly by hydrogen-bond interaction with the phosphate groups of DPC lipids. At 20 ns, the interaction of the α-helical segment with DPC micelle was contributed by the electrostatic interaction of the charge residue K17 and hydrogen-bond interactions between residues I9, T10, and the phosphate groups of the lipids. The C-terminal residues, I18, Y19, and W20 were bound to micelle by hydrogen-bond and hydrophobic interactions. However, the N-terminus is flexible and not bound. At 40 ns, the overall structure of GW-Q6 was entirely bound to the surface of DPC micelles. Especially, the electrostatic interaction of K6 to phosphate groups of lipid mainly contributed to the adherence of N-terminus to DPC micelle. This completely bounded conformation of GW-Q6 further embedded and penetrated the surface of micelle at 50 ns. Meanwhile, the α-helical segment and C-terminus, covered by DPC lipids, reached close to the interior of micelle. At 100 ns, GW-Q6 bound to an apparently distorted DPC micelle with the buried middle α-helical segment moderately exposing the N-terminus and C-terminus (Y19, and W20). The interaction between GW-Q6 and DPC micelle was also quantified by monitoring the distance between the center of mass of the peptide backbone and the center of mass of the micelle acyl chains as a function of simulation time (Fig 7C).

## Discussion

Small peptides generally exert their antimicrobial functions by binding and disrupting the integrity of the microbial membrane [7,42,43]. The amphipathic helical peptides interact with the negatively charged and lipidic constituents of the bacterial membrane via its cationic and hydrophobic residues [18,44]. A previous study suggested that the lipopolysaccharide (LPS) is the initial AMP-binding site on Gram-negative bacteria [28]. However, the exact role of LPS in the bactericidal activity of AMP remains unclear. Various mechanisms of action and targets of AMPs have been proposed and investigated including the inner membrane proteins, nucleic acids, intracellular proteins, outer surface lipids, and outer membrane proteins [8,45–47]. Our previous study indicates that the OprI (outer membrane protein I) of *P*. *aeruginos* is responsible for its susceptibility to human ribonuclease 7 (hRNase 7) and natural α-helical cationic AMPs instead of surface lipopolysaccharides. In this report, we show that a synthetic peptide, GW-Q6, may also exert its bactericidal activity by targeting the OprI receptor.

The dye leakage experiments showed the hierarchy of membrane-disruptive activity of the GW-Q6 peptide against the tested LUVs as POPG > POPC/POPG (3:1) > POPC, which is comparable to a previous report of DOPG > DOPC/DOPG (3:1) > DOPC [27]. These result suggest the selectivity of GW-Q6 against negatively charged bacterial membrane rather than mammalian cells. Intriguingly, ~2 μM of GW-Q6 can cause 100% dye leakage for purely neutral POPC LUVs. This is probably due to the hydrophobic portion of the peptide facilitating the amphipathic helix formation and enhancing the peptide-neutral vesicle interactions [27]. Consistently the strong membrane-disruptive ability of GW-Q6 is seen from its broad antimicrobial activity against Gram-negative bacteria such as *Klebsiella pneumoniae*, *Pseudomonas aeruginosa* ATCC27853, *P*. *aeruginosa* PAO1, and the Gram-positive *Listeria monocytogenes* (Fig 2A). On the other hand, the bactericidal activity of GW-Q6 against *P*. *aeruginosa* PAO1 was repressed with the addition of exogenous recombinant OprI to compete for GW-Q6 or anti-OprI antibodies to block surface OprI (Fig 3A). These observations demonstrate the potential interaction between the GW-Q6 peptide and the OprI receptor. The EDC cross-linking assay along with the Western blotting experiments showed that GW-Q6 probably targets OprIs on the surface of the bacteria with the loss of OprI recognition by anti-OprI antibody (Fig 3B–3D). GW-Q6 peptide targeted OprI on the surface of the bacteria and was then internalized into the cytosol leading to the susceptibility of *P*. *aeruginosa* to GW-Q6. Similar to most AMPs, the GW-Q6 peptide caused depolarization of the membrane potential and an increase of membrane permeability above the bactericidal concentrations of 4 μM for 10^2^-fold reduction in cfu (Fig 2B–2C). However, hyperpolarization was transiently observed for 1 min after the addition of GW-Q6 at sub-lethal concentrations of 1–2 μM. Although depolarization of membrane potential is considered to be an initial event of membrane injury, hyperpolarization is reported as an adaptation prior to bacterial cell death or full recovery depending on the concentration of peptides employed [48,49]. Hyperpolarization has also been associated with the formation of superoxide radicals which are involved in membrane integrity and cell viability [48,50].

Our CD experiments demonstrated that the GW-Q6 peptide has random coiled characteristics in PC buffer. Conversely, in negatively charged SDS micelles and zwitterionic DPC micelles solutions, the GW-Q6 peptide showed characteristics of α-helical structure (S1 Fig) demonstrating an induced disorder-to-ordered conformational transition upon binding to phospholipid membranes. This observation is consistent with previous studies where AMPs are disordered in aqueous conditions but become structured upon interaction with detergent micelles or phospholipid membranes [18,51–55]. This conformational change indicates that the α-helical structure is the active conformation capable of penetrating the bacterial membrane. As well, it is noteworthy that the binding of OprI to the GW-Q6 peptide is enhanced in the presence of 0.075% (2.6 mM) sarkosyl (Fig 4A). The sarkosyl-induced α-helical conformation may contribute to the enhanced binding ability of the GW-Q6 peptide to OprI (Fig 4B). The ANS assay showed the increased hydrophobicity of GW-Q6 induced by sarkosyl underlying the enhancement of interaction to OprI (Fig 4C). As a result, we propose that GW-Q6 may target OprI or fuse with bacterial membrane after being triggered by sarkosyl-like surfactant having an amphiphilic structure with a hydrophobic surface and anionic charge, and exert its antimicrobial activity.

With respect to the characteristics of surfactants, it is known that both anionic surfactant (SDS, sarkosyl) and non-ionic surfactant (Triton X-100, Tween 20) are employed to solubilize or extract proteins from inclusion bodies or membrane fraction of tissues at higher concentration, however they are also used to protect or stabilize proteins from urea- and thermal-induced denaturation. Currently, some anionic bio-surfactants, like sarkosyl, containing amide group are frequently used in industry making small-particle emulsions for cosmetics. The amide group connects the hydrophobic tail and the polar anionic head-group. These surfactants could self-assemble into polymers in the form of monomer, micelle, interdigitated or fully developed bilayer depending on the concentrations of surfactant and counter ions in the solution. The CMC value (critical micelle concentration) of sarkosyl in 20 mM phosphate, pH7.9, is 6.2 mM which is less than that obtained in pure water, 13 mM, due to increased presence of counter ion (Na^+^). As the concentration of sarkosyl in 20 mM phosphate, pH7.9, reaches 5 mM, it starts to destroy the α-helical structure of bovine serum albumin and completely disrupts the protein structure at 12 mM [56,57]. The concentration of sarkosyl (0.075%, 2.6mM) employed in this study was able to induce α-helix formation and bind to OprI, which may be lower than the reported CMC values (6 mM and 13 mM for phosphate buffer and pure water, respectively). It is suggested that the amphiphilic sarkosyl may self-assemble and provide a membrane-like structure, no matter monolayer, bilayer or micelle, for AMP to anchor or fuse. Although the 0.075% sarkosyl was able to promote the formation of α-helix and decrease the viability of *Pseudomonas aeruginosa* by itself, further studies on its cytotoxicity to mammalian cells are prerequisite to develop as an enhancer for anti-infective agents.

The structural basis for the GW-Q6 peptide’s mode of action against microbes was investigated by ^1^H NMR in the presence of DPC micelles. The well-dispersed signals of 2D-NOESY and TOCSY (Fig 5A and 5B) aligned with the CD result showed that the GW-Q6 peptide has a well-defined α-helical structure in DPC micelles. The amino residues K7-K17 of the amphipathic GW-Q6 peptide assumes an apparent α-helical structure rich in positively charged side chains (K3, K7, K13, K14, and K17) that attract the negatively charged bacterial membrane. As reported by *Chou et*.*al*, the α-helicity of the GW-Q6 peptide is highly correlated with the dye-leakage capability and antibacterial activity [27]. Therefore, this α-helical segment has been suggested to play an essential role in membrane permeabilization and antimicrobial activity. Moreover, three GW-Q6 analogues, GW-Q3, GW-Q4 and GW-Q5, possess the same polar angle, hydrophobicity, and hydrophobic moment but differ in their charges (GW-Q3 is +3, GW-Q4 is +4, GW-Q5 is +5, and GW-Q6 is +6). The multiple sequence alignment analysis showed that four lysine residues (K6, K7, K13, and K14) are highly conserved among peptides (S2 Fig). Structurally, K7, K13, and K14 are located at the α-helical segment with K13 and K14 involved in electrostatic interaction with DPC micelles. This observation with MD simulation suggests that these conserved lysine residues are crucial for helical formation and highly associated with membrane binding ability.

Previously we reported that OprI mainly contributes to the susceptibility of *P*. *aeruginosa* to hRNase and cationic α-helical AMPs, but not other AMPs with distinct secondary structures [28]. The cationic α-helical AMP, SMAP-29, consists of an N-terminal flexible domain (^1^RGLRRLG^7^) in front of the central α-helix (residues 8–17) and a C-terminal hydrophobic segment (residues 20–28). This N-terminal flexible region is followed by a rigid α-helix which exerts bactericidal activity in *P*. *aeruginosa* through the receptor OprI. Likewise, the structure of GW-Q6 is composed of a flexible N-terminus (^1^GIKIAK^6^) and an α-helical segment (K7-K17). This similarity reasonably correlates with the structure-function relationship of GW-Q6 to the OprI receptor. Apart from interacting with receptors on the surface of bacteria, most amphipathic helices of antimicrobial peptides are prone to bind directly to the interfacial region of bacterial membranes in such an orientation with its hydrophobic face embedded in the membrane while the polar region is exposed to the solvent [58–63]. Our NMR paramagnetic probe studies with 5-, 12-, 16-DSA, and Mn^2+^ ions (Fig 6) revealed that the GW-Q6 peptide orients itself into DPC micelles with the N-terminus (residue I2 and K3) near the head group of lipids and the C-terminus (residue K17-W20) outside or at the surface of the micelles. While the α-helical segment (residues K7-K17) of GW-Q6 is more buried, possibly with the positively charged residues (K3, K6, K7, K13, K14, and K17) facing the surface of micelle and the hydrophobic residues oriented toward the micelle’s interior. Further MD simulation demonstrates that hydrogen bond and/or electrostatic interactions are the major driving forces for the initial approximations of GW-Q6 to micelle. During the course of 10–20 ns, the transitional α-helical segment (I11-K17) and the C-terminus first anchor onto the micelle. Meanwhile, the aromatic residues Y19 and W20 position the C-terminus of GW-Q6 on the surface of the micelle and cooperatively assist the α-helical segment to bind to the micelle. Additionally, the lysine residues (K13, K14, and K17) of the α-helical segment stabilize the interaction with micelle by electrostatic attractions. Consequently, this α-helical segment penetrated and buried into the micelle, whereas residues Y19 and W20 of the C-terminus were more exposed to the solvent [64], This observation is comparable with our PRE result and again reveals the biological function of this α-helical segment in membrane disruption and antimicrobial activity. However, the unstructured N-terminus freely moved at the early stage of the simulation, but finally stabilized when bound to the surface of the micelle with residues I4-K7 more solvent exposed than those observed in the PRE experiments. This inconsistency is reasonable since both NMR and MD simulations have their individual limitations. The NMR results did reveal the structure and orientation of the GW-Q6 peptide with reliable precision. The MD simulation can capture the hidden conformation and behavior of the GW-Q6 peptide upon binding to DPC micelles which may not be able to be discerned by NMR experiments. Therefore, by integrating the complementary findings from NMR and MD simulations, we proposed a feasible and biologically functional structure of GW-Q6 in DPC micelles as presented in Fig 6C.

## Conclusion

The synthetic peptide GW-Q6 may exert antimicrobial activity against P. *aeruginosa* by targeting the outer membrane protein OprI. The binding of GW-Q6 to OprI is strongly enhanced by sarkosyl which alters the secondary structure of GW-Q6 to become α-helical with increased hydrophobicity. GW-Q6 orients itself into DPC micelles with its N-terminus (I2 and K3) near the head group of the lipids, its central α-helix segment (K7-K17) buried, and its C-terminus (K17-W20) outside the DPC micelle. The α-helical segment (K7-K17) of GW-Q6 is critical for membrane permeabilization and antimicrobial activity. The conserved lysine residues K3, K7, and K14 are essential for helix formation and membrane binding affinity. The aromatic residues Y19 and W20 are functionally important in positioning the C-terminus on the surface of micelles during the early stages. Our study provides insight into the possible mechanism of action and residue-specific annotation of the GW-Q6 peptide and suggests its applicability in developing new anti-infective agents.

## Supporting Information

S1 Fig CD spectra of GW-Q6 in buffer, TFE, SDS and DPC. (TIF)

S2 Fig Multiple sequence alignment of GW-Q6 and its analogues. (TIF)

S1 File Paramagnetic relaxation enhancement (PRE) experiments and Molecular Dynamics Simulation. (PDF)
