# Supplementary figures and images for: Sarkosyl-Induced Helical Structure of an Antimicrobial Peptide GW-Q6 Plays an Essential Role in the Binding of Surface Receptor OprI in Pseudomonas aeruginosa
Source: PLoS One. 2016 Oct 11;11(10):e0164597. doi: 10.1371/journal.pone.0164597 (PMC5058510; doi:10.1371/journal.pone.0164597)

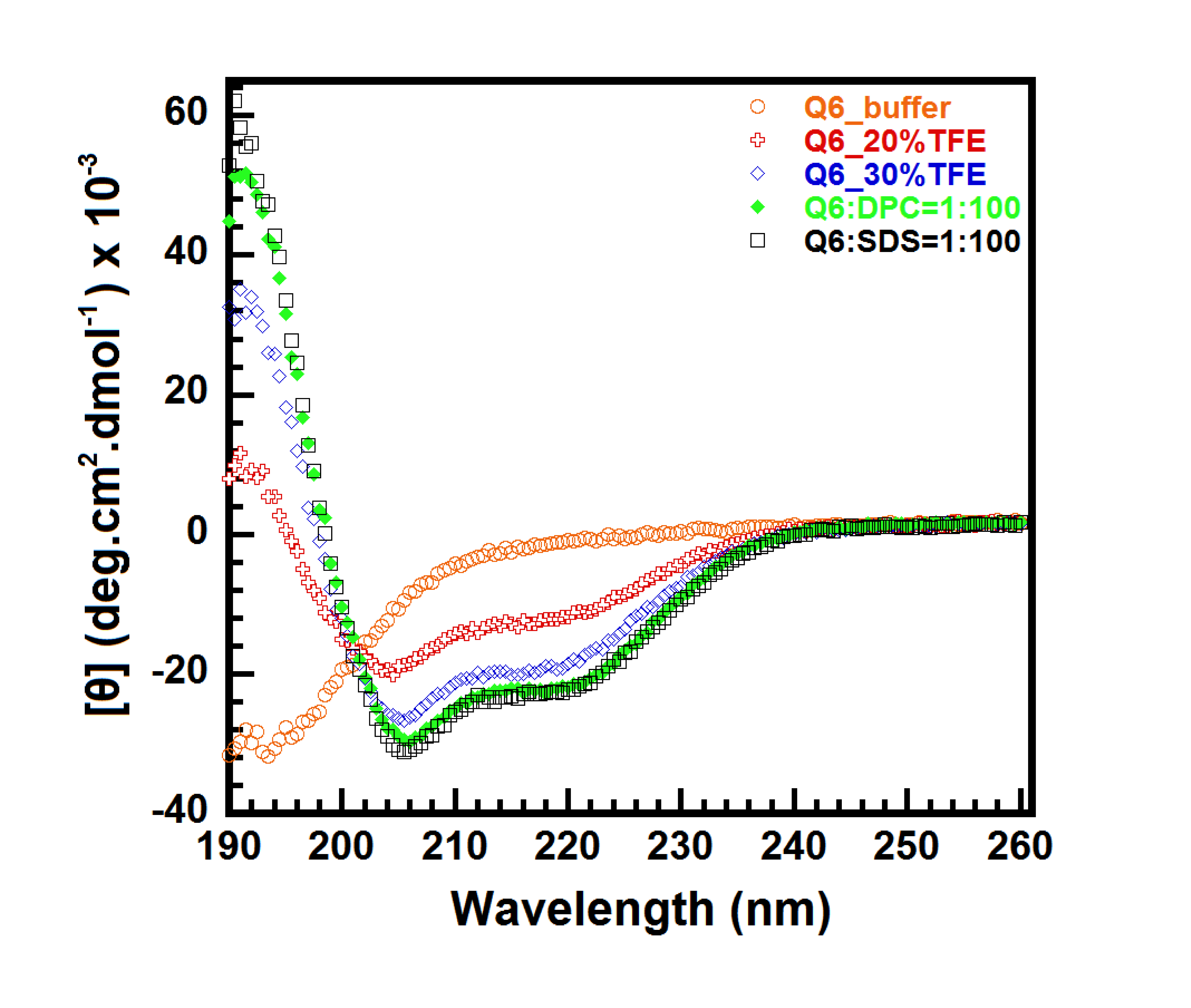

Supplement: S1 Fig — (TIF) [file pone.0164597.s001.tif]

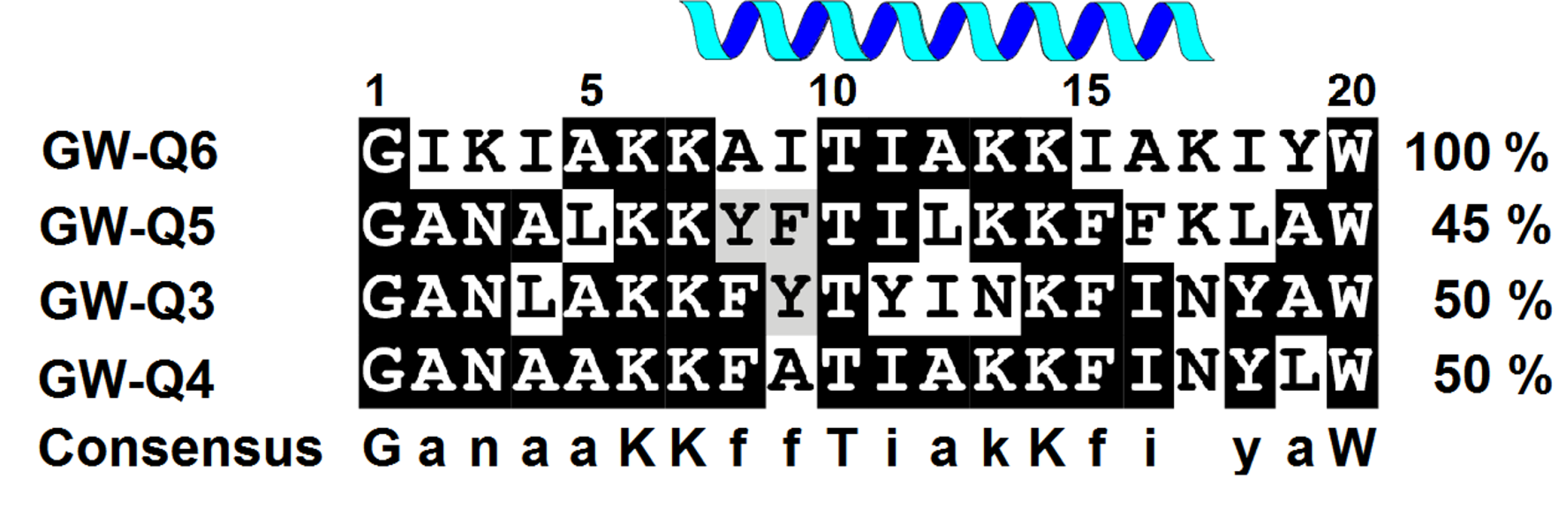

Supplement: S2 Fig — (TIF) [file pone.0164597.s002.tif]
