# Supplementary material for: Sarkosyl-Induced Helical Structure of an Antimicrobial Peptide GW-Q6 Plays an Essential Role in the Binding of Surface Receptor OprI in Pseudomonas aeruginosa
Source: PLoS One. 2016 Oct 11;11(10):e0164597. doi: 10.1371/journal.pone.0164597 (PMC5058510; doi:10.1371/journal.pone.0164597)
Supplement: S1 File — (PDF) [file pone.0164597.s003.pdf]

Sarkosyl-induced helical structure of an antimicrobial peptide GW-Q6 plays an essential role in the binding of surface receptor OprI in *Pseudomonas aeruginosa*

Tien-Sheng Tseng[1], Shih-Han Wang[1], Ting-Wei Chang[1], Hung-Mu Wei[1], I-June Wang[1], Keng-Chang Tsai[2,3], You-Di Liao[1] and Chinpan Chen[1*]*

[1]Institute of Biomedical Sciences, Academia Sinica Taipei 115, Taiwan

[2]National Research Institute of Chinese Medicine, Ministry of Health and Welfare, Taipei 112, Taiwan.

[3]The Ph.D. Program for Medical Biotechnology, College of Medical Science and Technology, Taipei Medical University, Taipei 110, Taiwan.

*Corresponding authors:

You-Di Liao, Ph.D.; Phone: +886-2-26523053 Fax: +886-2-27829142; E-mail address: ydliao@ibms.sinica.edu.tw

Chinpan Chen, Ph. D.; Phone: +886-2-26523035; Fax: +886-2-27887641; E-mail address: bmchinp@ibms.sinica.edu.tw

Supporting Information

Methods

Paramagnetic relaxation enhancement (PRE) experiments

The paramagnetic relaxation enhancement (PRE) experiments were conducted with the addition of aliquots of Mn^{2+}, 5-doxyl-stearic acid (5-DSA), 12-doxyl-stearic acid (12-DSA), and 16-doxyl-stearic acid (16-DSA) in the NMR sample. Mn^{2+} ions solution (0.1M) was prepared by dissolving $MnCl_2 \cdot 4H_2O$ in 10 mM sodium phosphate pH 5.0 buffer. For paramagnetic relaxation enhancement effect of Mn^{2+} ions, GW-Q6 peptide was firstly mixed with DPC micelles (molar ratio = 1:100) and then vortexed for 3-5 min and incubating at room temperature for 10 min. After that, Mn^{2+} ions were added to reach the final concentration of 0.1, 0.5, and 1 mM in GW-Q6 (1.5 mM)-micelle solution, and equilibrated for 15 mins before recording ^1H-^1H TOCSY spectra. For paramagnetic relaxation enhancement effect, the DAS solutions were prepared in deuterated methanol (D_4-MeOH). The DSA was firstly mixed with DPC (in 10 mM sodium phosphate, pH 5.0) with a molar ration of 1:60. Subsequently, peptide GW-Q6, buffered in 10 mM sodium phosphate, pH 5.0, was added to the prepared DSA-DPC mixed micelle solution. The final concentrations of DSA, DPC and GW-Q6 were 2.5, 150, and 1.5 mM, respectively, and these NMR samples were equilibrated for 15 min before ^1H-^1H TOCSY spectra were recorded. All ^1H-^1H TOCSY spectra were acquired at 320 K with 2048 data points in t_2 and 320 points in t_1. The cross peak intensities of peptide GW-Q6 were measured with and without paramagnetic solutions and calculated based on established protocols [1].

Molecular Dynamics Simulation

The molecular dynamics simulations were conducted using the GROMACS software package, version 4.6.7. [2-4]. The GROMOSE96 force field parameters were employed for the peptide and solvent. The GROMOS united-atom parameter set, lipid.itp, and the pre-equilibrated micelles (65 DPC molecules) with 6305 water molecules were downloaded from Tieleman laboratory (http://moose.bio.ucalgary.ca). Our determined solution structure of GW-Q6 was used as the starting conformation for the simulation. GW-Q6 was initially placed at the DPC-water interface. The peptide-micelle system was placed in a 54 $Å^3$ periodic box of spc216 water. 47 chloride and 41 sodium counter ions were added as 0.1 mM electrolyte. GW-Q6 was positionally constrained and subjected to conjugated gradient minimization to eliminate interfering contacts [5]. The simulation was thermostatted at 310 K [6,7] and the temperature was coupled with Berendsen algorithm (coupling constant = 0.1 ps) [8]. The calculation of electrostatic interactions and the cutoff for van der Waals interactions were conducted with particle mesh Ewald algorithm [9]. The steepest decent algorithm was used for energy minimization down to a maximum gradient of 1000 kJ $mol^{-1}nm^{-1}$. NVT-ensemble state conduction was used for equilibrium, in which heavy atoms of peptide were positionally restrained by applying a spring constant of 1000 kJ $mol^{-1}nm^{-2}$. Additionally, all bonds were constrained with the LINCS algorithm [10]. After that, the isothermal-isobaric (NPT) equilibration was performed for 10,000 ps with Parrinello-Rahman algorithm (coupling constant = 0.5 ps) [11]. The position restraint used in NPT was also employed in NPT equilibration. Furthermore, the molecular dynamics simulation was carried out for 100 ns with an NPT ensemble.

Figures

S1 Fig

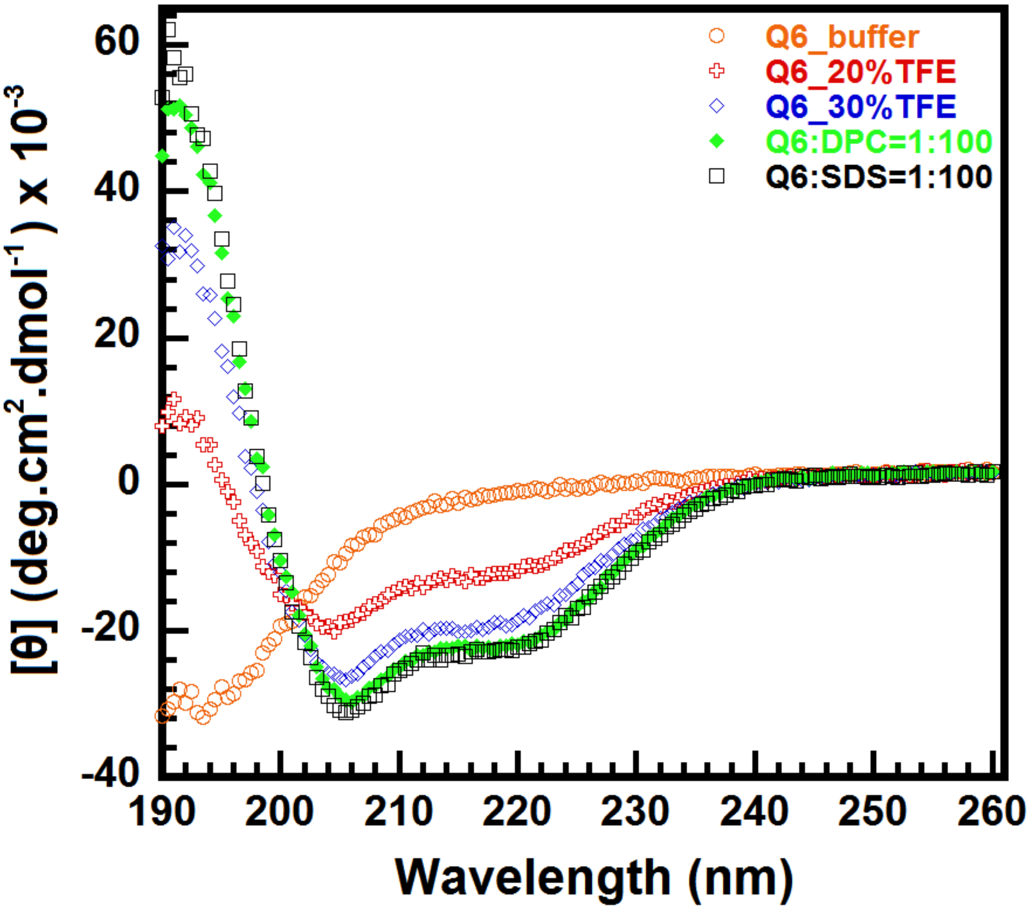

S1 Fig. CD spectra of GW-Q6 in buffer, TFE, SDS and DPC.

S2 Fig

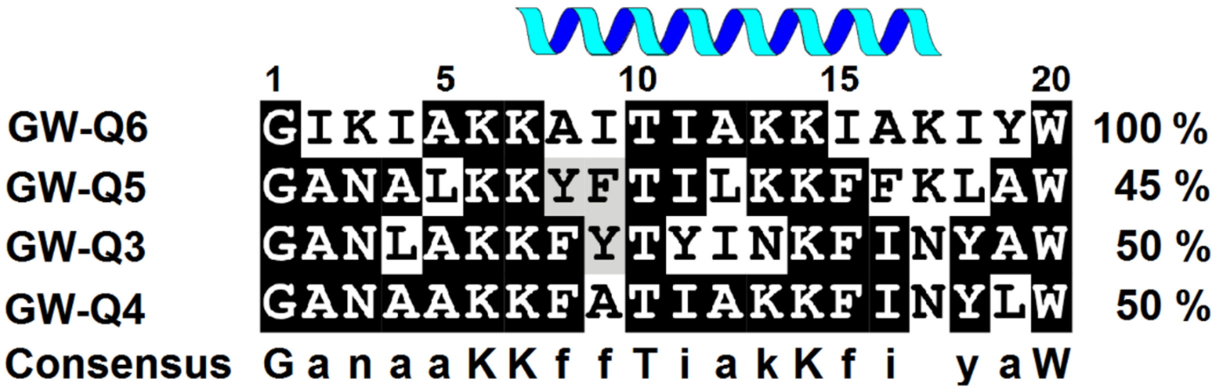

S2 Fig. Multiple sequence alignment of GW-Q6 and its analogues.

The multiple sequence alignment of GW-Q6 and its analogues (GW-Q3, GW-Q4 and GW-Q5) was conducted using the T-coffee algorithm and the on-line server ExPASy (www.uniprot.org/). The identical, conserved and semi-conserved residues are shaded in black, dark gray and pale gray, respectively. In the consensus sequence, capital and small letters are used to indicate those residues found in all or most of the sequences, respectively.

References

1. Bhunia A, Mohanram H, Bhattacharjya S (2012) Structural determinants of the specificity of a membrane binding domain of the scaffold protein Ste5 of budding yeast: implications in signaling by the scaffold protein in MAPK pathway. Biochim Biophys Acta 1818: 1250-1260.
2. Van Der Spoel D, Lindahl E, Hess B, Groenhof G, Mark AE, et al. (2005) GROMACS: fast, flexible, and free. J Comput Chem 26: 1701-1718.
3. Hess B, Kutzner C, van der Spoel D, Lindahl E (2008) GROMACS 4: Algorithms for Highly Efficient, Load-Balanced, and Scalable Molecular Simulation. J Chem Theory Comput 4: 435-447.
4. Pronk S, Pall S, Schulz R, Larsson P, Bjelkmar P, et al. (2013) GROMACS 4.5: a high-throughput and highly parallel open source molecular simulation toolkit. Bioinformatics 29: 845-854.
5. Gordon LM, Mobley PW, Lee W, Eskandari S, Kaznessis YN, et al. (2004) Conformational mapping of the N-terminal peptide of HIV-1 gp41 in lipid detergent and aqueous environments using 13C-enhanced Fourier transform infrared spectroscopy. Protein Sci 13: 1012-1030.
6. Tieleman DP, Forrest LR, Sansom MS, Berendsen HJ (1998) Lipid properties and the orientation of aromatic residues in OmpF, influenza M2, and alamethicin systems: molecular dynamics simulations. Biochemistry 37: 17554-17561.
7. Zhao W, Rog T, Gurtovenko AA, Vattulainen I, Karttunen M (2007) Atomic-scale structure and electrostatics of anionic palmitoyloleoylphosphatidylglycerol lipid bilayers with Na+ counterions. Biophys J 92: 1114-1124.
8. Berendsen HJC, Postma JPM, van Gunsteren WF, Di Nola A, Haak JR (1984) Molecular dynamics with coupling to an external bath. J Chem Phys 81: 3684-3690.
9. Essmann U, Perera L, Berkowitz ML, Darden, T, Lee H, Pedersen LG (1995) A smooth particle mesh Ewald method. J Chem Phys 103: 8577-8592.
10. Hess B, Bekker H, Berendsen HJC & Fraaije JGEM (1997) LINCS: A linear constraints solver for molecular simulation. J Comput Chem 18: 1463-1472.
11. Parrinello M, Rahman, A. (1981) Polymorphic transitions in single crystals: A new molecular dynamics method. J Appl Phys 52: 7182-7190.
